# Comprehensive Morphometric MRI Assessment in Children with Breath-Holding Spells: Integration of Automated (Vol2Brain) and Semi-Automated (3D Slicer) Segmentation Methods

**DOI:** 10.3390/tomography12020021

**Published:** 2026-02-06

**Authors:** Adil Aytaç, Hilal Aydın

**Affiliations:** 1 Department of Radiology, Faculty of Medicine, Health Practice and Research Hospital, Balikesir University, 10145 Balıkesir, Turkey; 2 Department of Pediatric Neurology, Faculty of Medicine, Health Practice and Research Hospital, Balikesir University, 10145 Balıkesir, Turkey; drhilalaydin@gmail.com

**Keywords:** pediatric, magnetic resonance imaging, neuroimaging

## Abstract

Objectives: To evaluate regional anatomical differences in brain volume, surface area, and cortical thickness between children with breath-holding spells (BHSs) and a control group using morphometric MRI analyses. Methods: Three-dimensional T1-weighted cranial MRI data from 48 children with BHSs and 50 control children were retrospectively analyzed, yielding volumetric, surface area, and cortical thickness measures for 135 brain regions. All measurements were assessed relative to total intracranial volume (ICV). Group comparisons were performed using analysis of covariance with age, sex, and ICV as covariates, followed by Benjamini–Hochberg false discovery rate correction (q < 0.05). Results: The BHS group exhibited reduced bilateral amygdala volumes (left: q = 0.042; right: q = 0.038). Both cortical thickness and volume were reduced in the right anterior insula (thickness: q = 0.046; volume: q = 0.049). In addition, cortical thickness was reduced in the bilateral anterior cingulate cortices (left: *p* = 0.019, q = 0.045; right: *p* = 0.017, q = 0.043) as well as in the right medial frontal cortex (*p* = 0.009, q = 0.036). Subregional cerebellar analysis demonstrated volume reductions in the right lobule VI (q = 0.031), left lobule VIIA (Crus I) (q = 0.043), and vermis IX–X (q = 0.039). Conclusions: Detecting measurable morphometric changes in brain regions involved in autonomic and emotional regulation in children with BHSs will contribute to understanding the neurobiological characteristics associated with BHSs.

## Simple Summary

This study is among the first to comprehensively evaluate regional brain and cerebellar morphology in children with breath-holding spells (BHSs) by integrating automated (Vol2Brain) and semi-automated (3D Slicer) segmentation methods. Our findings demonstrated significant morphometric differences in the bilateral anterior cingulate cortices, right anterior insular cortex, bilateral amygdalae, right medial frontal cortex, and some cerebellar subregions in children with BHSs. These results suggest that measurable morphometric differences in certain anatomical structures related to the limbic and autonomic systems may be associated with BHSs.

## 1. Introduction

Breath-holding spells (BHSs) are benign paroxysmal episodes characterized by brief apnea, cyanosis, and transient loss of consciousness in response to an emotional or painful stimulus [1]. Population-based studies report a prevalence of approximately 4–5%, with no significant sex difference [1,2]. The incidence peaks between 1 and 3 years of age, and in most cases, the episodes spontaneously resolve by school age [2]. Clinically, BHSs are classified into two subtypes: cyanotic and pallid [1]. The cyanotic type typically occurs during the expiratory phase following anger or frustration and is accompanied by perioral cyanosis. In contrast, the pallid type is triggered by sudden fright or minor trauma and manifests as a brief syncope-like collapse. Although the clinical presentation may appear dramatic, BHSs are generally considered neurologically benign and self-limiting events without lasting sequelae [1,2].

The pathophysiology of BHSs is linked to a transient disruption of the sympathetic–parasympathetic balance within the central autonomic network [3]. This network encompasses complex interactions among the prefrontal cortex, anterior cingulate gyrus, insula, amygdala, hypothalamus, brainstem nuclei, and cerebellar structures [4]. Following an emotional or nociceptive trigger, exaggerated vagal reflex activation and temporary inhibition of the brainstem cardiorespiratory centers result in bradycardia, apnea, and transient cerebral hypoperfusion [5]. During this hemodynamic response, feedback loops between frontal cortical regions and cerebellar pathways play a pivotal role in modulating autonomic regulation and emotional responses. Functional MRI and structural connectivity studies demonstrate that the cerebellum contributes not only to motor coordination but also to the maintenance of cardiovascular and respiratory homeostasis [6]. In particular, the vermis and fastigial nuclei are thought to modulate vagal output through bidirectional connections with medullary autonomic centers [7]. Collectively, these findings suggest that the transient loss of consciousness and cyanosis observed in BHSs may arise from temporary dysfunction within the brainstem–cerebellar–frontal network. Consequently, the hypothesis that recurrent episodes of hypoperfusion and hypoxemia may exert subtle yet measurable morphometric effects on developing frontocerebellar autonomic circuits appears biologically plausible.

Despite the clinical prevalence of BHSs, the existence of specific structural brain variations associated with these episodes remains poorly understood. This scarcity of high-resolution morphometric data hampers the identification of anatomical correlates that may underpin autonomic dysregulation in BHS patients. While previous studies have discussed autonomic regulation and brainstem-level functional responses, quantitative analyses of cortical and cerebellar volumetric parameters have not yet been systematically explored [1,2,5]. Recent advances in brain segmentation and voxel-based morphometry have enabled the detection of microstructural alterations in various pediatric conditions associated with recurrent hypoperfusion or hypoxemia—such as febrile seizures, syncope, and sleep apnea—particularly within cerebellar, frontal, and limbic regions [8,9]. These findings support the notion that repetitive autonomic events may induce measurable effects on brain morphology [9,10].

Given the recognized role of the frontocerebellar network in emotional modulation and autonomic control, a comprehensive morphometric evaluation of global and region-specific brain structures may objectively reveal structural correlates secondary to BHSs [9,10]. Therefore, segmentation-based voxel-wise volumetric MRI analysis of children with BHSs could address a significant gap in the literature and represent one of the first quantitative investigations of potential brain morphological alterations associated with these episodes. However, early childhood is characterized by substantial interindividual variability in brain volumes—shaped by ongoing brain maturation and biological factors such as nutritional status and iron levels—accordingly, identifying BHS-associated structural patterns requires an analytical framework that accounts for developmental variability [1,2].

By integrating whole-brain and region-specific volumetric assessments, this study aims to provide quantitative neuroimaging data on brain morphology in BHSs and to contribute to the existing literature on the structural characteristics of this condition. Given the limited neuroimaging literature on BHSs, the study was designed as an initial exploratory investigation. Although specific brain regions were examined based on their established roles in autonomic regulation, the findings are interpreted as hypothesis-generating rather than confirmatory and are intended to inform future longitudinal and hypothesis-driven research.

## 2. Materials and Methods

### 2.1. Study Design and Participants

This retrospective, cross-sectional study was approved by the institutional ethics committee (date: 2 September 2025; Decision No: 2025/6-13) and conducted in accordance with the principles of the Declaration of Helsinki. A total of 48 children diagnosed with BHSs and 50 controls were enrolled and evaluated between January 2019 and July 2025. Inclusion and exclusion criteria were strictly defined to maintain cohort homogeneity and isolate potential morphometric variations associated with BHSs. The age range of 6 months to 6 years was selected to align with the peak clinical incidence of BHSs. By excluding comorbid neurological conditions and implementing rigorous image quality controls, we minimized confounding factors that could independently influence neurodevelopmental trajectories.

Inclusion Criteria:

BHS group:

- Age between 6 months and 6 years;
- Clinical diagnosis of BHSs established by a pediatric neurologist;
- Normal neurodevelopmental status and neurological examination findings;
- Availability of diagnostic-quality 3D T1-weighted cranial MRI;
- Absence of pathological findings on MRI;
- MRI performed at the same institution.

Control group:

- Age between 6 months and 6 years;
- Underwent cranial MRI at the same institution for benign clinical indications such as headache, dizziness, or syncope, with no abnormal neurological findings;
- Normal neurological examination;
- No history of epilepsy, chronic systemic disease, or regular medication use;
- MRI performed at the same institution.

All control MRI examinations were performed for transient, non-specific neurological complaints and were reviewed by experienced neuroradiologists. Children with any structural, developmental, or signal abnormalities on MRI, or with abnormal neurological examination findings, were strictly excluded. This approach was adopted to ensure ethical feasibility and to provide a clinically screened reference group with structurally normal brain MRI, which is commonly used in pediatric neuroimaging studies.

Exclusion Criteria (for both groups):

- History of psychiatric disorder;
- Presence of a chronic disease requiring long-term medication;
- History of known vascular or demyelinating disease;
- Cranial MRI obtained outside the institution;
- Incomplete clinical or imaging data;
- Detection of epileptogenic foci or structural brain lesions on MRI;
- Poor image quality rendering MRI non-diagnostic.

The diagnosis of BHSs was established by an experienced pediatric neurologist based on clinical observations and characteristic episode features. Data regarding attack frequency, duration, triggering factors, and spell subtype were retrospectively retrieved from the hospital information system.

### 2.2. Magnetic Resonance Imaging Protocol

All examinations were performed using a standardized cranial MRI protocol on a 1.5 Tesla scanner (Ingenia, Philips Medical Systems, Best, The Netherlands). The detailed MRI sequence parameters used in the epilepsy protocol were as follows:

- Axial T1-weighted spin-echo (SE): Repetition time/echo time (TR/TE) 450/15 ms, field of view (FOV) 230 mm, slice thickness 5 mm, matrix 308 × 183.
- Axial fat-saturated T1-weighted SE: TR/TE 633/15 ms, FOV 230 mm, slice thickness 5 mm, matrix 308 × 183.
- Axial T2-weighted turbo SE: TR/TE 5240/100 ms, FOV 230 mm, slice thickness 5 mm, matrix 384 × 237.
- Coronal fluid-attenuated inversion recovery (FLAIR): TR/TE 11,000/130 ms, FOV 230 mm, slice thickness 5 mm, matrix 256 × 157.
- Coronal T2-weighted turbo SE: TR/TE 3027/100 ms, FOV 200 mm, slice thickness 3 mm, matrix 336 × 217.
- Coronal T1-weighted inversion recovery: TR/TE 3079/15 ms, FOV 200 mm, slice thickness 3.5 mm, matrix 336 × 211.
- Coronal FLAIR: TR/TE 11,000/130 ms, FOV 230 mm, slice thickness 3 mm, matrix 256 × 157.
- Three-dimensional (3D) FLAIR: TR/TE 4800/315 ms, FOV 250 mm, slice thickness 1.04 mm, matrix 216 × 218.
- Axial diffusion-weighted imaging: b-values 0 and 1000 s/mm^2^, FOV 230 mm, slice thickness 5 mm, matrix 152 × 106.
- Axial 3D T1-weighted MRI: TR/TE 2500/46 ms, FOV 230 mm, slice thickness 1.04 mm, matrix 256 × 256.

The detailed MRI sequence parameters used in the routine brain protocol were as follows:

- Axial T1-weighted spin-echo (SE): TR/TE = 450/15 ms; FOV = 230 mm; slice thickness = 5 mm; matrix = 308 × 183.
- Axial fat-saturated T1-weighted SE: TR/TE = 633/15 ms; FOV = 230 mm; slice thickness = 5 mm; matrix = 308 × 183.
- Axial T2-weighted turbo SE: TR/TE = 5240/100 ms; FOV = 230 mm; slice thickness = 5 mm; matrix = 384 × 237.
- Coronal fluid-attenuated inversion recovery (FLAIR): TR/TE = 11,000/130 ms; FOV = 230 mm; slice thickness = 5 mm; matrix = 256 × 157.
- Coronal T1-weighted inversion recovery: TR/TE = 3079/15 ms; FOV = 200 mm; slice thickness = 3.5 mm; matrix = 336 × 211.
- Axial diffusion-weighted imaging (DWI): b-values = 0 and 1000 s/mm^2^; FOV = 230 mm; slice thickness = 5 mm; matrix = 152 × 106.
- Axial 3D T1-weighted MRI: TR/TE = 2500/46 ms; FOV = 230 mm; slice thickness = 1.04 mm; matrix = 256 × 256.

No contrast agent was administered in any of the cases. Due to the absence of pediatric intensive care support in our center, cranial MRI could not be conducted under anesthesia or sedation. Instead, all imaging procedures were completed during spontaneous natural sleep, following routine scheduling and parental guidance. Patients with BHSs were examined using the epilepsy MRI protocol because these children were referred for neuroimaging to exclude epileptic seizures or other paroxysmal neurological conditions in accordance with routine pediatric neurology practice. In contrast, the control group was examined using the routine brain MRI protocol, as imaging was performed for benign, non-epileptic clinical indications, and the application of an extended epilepsy protocol was not clinically indicated. The axial 3D T1-weighted MRI sequence (TR/TE = 2500/46 ms; FOV = 230 mm; slice thickness = 1.04 mm; matrix = 256 × 256), which constituted the sole input for volumetric analysis, was acquired with identical imaging parameters in both protocols. Therefore, minor variations in other sequences between protocols did not introduce heterogeneity in the quantitative morphometric analyses, as no additional sequences were used for volumetric measurements. Throughout the study period (2019–2025), the 1.5 Tesla MRI system operated under a stable hardware configuration and a consistent software environment. Retrospective review of the technical logs confirmed that no modifications to gradient coils, radiofrequency chains, or reconstruction algorithms were implemented that might have influenced the signal-to-noise ratio or T1-weighted contrast characteristics. Consequently, this single-center and single-scanner design provided a uniform technical framework, where all 3D T1-weighted acquisitions were performed using identical hardware components and reconstruction pipelines, thereby minimizing potential bias from temporal measurement drift or system-related heterogeneity.

### 2.3. Morphometric Brain Analysis Using Segmentation-Based Methods

#### 2.3.1. Vol2Brain-Based Automated Segmentation and Quantitative Analysis

MRI data were processed using Vol2Brain (version 1.0, release 23 November 2021, Universitat Politècnica de València, Valencia, Spain), a fully automated volumetric analysis platform [11]. Operating on the SPM12 and CAT12 frameworks, this system employs high-resolution voxel-based morphometry and volumetric quantification algorithms [12,13]. These tools enable standardized processing of T1-weighted data in both DICOM and NIfTI formats, ensuring consistent segmentation across the cohort.

All MRI datasets were retrieved in DICOM format from the institutional archive. To ensure optimal segmentation accuracy, only high-resolution 3D T1-weighted sequences with isotropic voxel sizes ≤ 1 mm^3^ were included [14]. Prior to analysis, all scans were visually screened by a board-certified neuroradiologist with nine years of experience for motion artifacts, field inhomogeneity, and anatomical completeness. Only datasets meeting predefined diagnostic quality criteria were accepted. Subsequently, DICOM datasets were anonymized and converted to NIfTI format using dcm2niix, as implemented in the MRIcroGL software package (Chris Rorden, University of South Carolina, Columbia, SC, USA) [15]. The retrospective design of the study inherently limited prospective randomization. All three-dimensional T1-weighted MRI datasets were inspected prior to segmentation to identify motion-related artifacts. In cases of excessive motion, post-processing motion correction algorithms—primarily based on rigid-body realignment and slice-to-volume interpolation techniques—were applied for quality assessment. However, because such procedures may introduce interpolation-related bias and compromise voxel-wise signal integrity in voxel-based morphometric analyses, datasets requiring post-processing motion correction were excluded. Accordingly, 7 patients in the BHS group and 10 patients in the control group were excluded due to motion artifacts or the application of post-processing motion correction. Only datasets that met predefined image quality criteria without requiring post-processing correction were included in the final analysis. Verified datasets were then uploaded to the secure Vol2Brain server for automated processing [11,16].

The Vol2Brain analysis pipeline consists of a sequence of fully automated processing steps. Initially, images are spatially normalized to the Montreal Neurological Institute (MNI152) template and corrected for bias-field inhomogeneity. Non-brain tissues, including the skull, scalp, and meninges, are subsequently removed using deformation-based surface modeling. Voxel-wise probabilistic algorithms are then applied to segment gray matter (GM), white matter (WM), and cerebrospinal fluid (CSF).

Cortical and subcortical parcellations are performed using a non-local multi-atlas patch-based approach implemented within the Vol2Brain pipeline [11]. Although volumetric results are reported according to the Neuromorphometrics, Harvard–Oxford (HOA/HOA2.0) [17], and Desikan–Killiany nomenclatures, the segmentation process relies on a library of multiple templates to adapt to individual neuroanatomical variability [12,13]. This approach is particularly suitable for pediatric cohorts aged 6 months to 6 years [6,7,8,10], as it minimizes systematic biases associated with single-atlas warping.

Following segmentation, the software automatically computes absolute and relative volumes (mm^3^ and %) for each anatomical structure. Within a high-resolution morphometric framework, Vol2Brain provides volumetric, surface-based, and cortical thickness metrics for 135 anatomical regions encompassing cortical, subcortical, cerebellar, brainstem, and CSF compartments [1,11,16].

At the cortical level, the platform delineates the frontal, parietal, temporal, occipital, cingulate, and insular cortices, including their respective subgyral and sulcal subdivisions. At the subcortical level, deep gray matter nuclei—including the caudate nucleus, putamen, globus pallidus, thalamus, nucleus accumbens, hippocampus, and amygdala—are quantitatively analyzed [11,16,17]. Ventricular structures (lateral, third, and fourth ventricles) as well as total intracranial volume (ICV) are also automatically measured. In addition, the analysis includes volumetric quantification of cerebellar hemispheres, vermian lobules, and cerebellar white matter.

Beyond regional volumetry, the software calculates mean cortical thickness (in mm) across all cortical regions, enabling assessment of hemispheric symmetry and regional morphometric variability, as described in prior morphometric studies [16,18,19]. This comprehensive and standardized segmentation workflow yields high-precision morphometric datasets and facilitates reproducible quantitative comparisons of global and regional brain morphology [11,14]. An example of an automated volumetric analysis output derived from the T1-weighted MRI scan of a pediatric patient with BHSs is presented in Supplementary Materials.

Supplementary Materials Automated volumetric analysis output generated by Vol2Brain from a T1-weighted MRI scan of a pediatric patient with BHSs. The image demonstrates color-coded segmentation of cortical, subcortical, and cerebellar structures, illustrating the detailed regional parcellation achieved through the fully automated voxel-based morphometric pipeline.

#### 2.3.2. Detailed Cerebellar Morphometric Analysis Using 3D Slicer

The integration of automated (Vol2Brain) and semi-automated (3D Slicer version 5.6.1; Brigham and Women’s Hospital, Boston, MA, USA) segmentation methods was strategically adopted to balance high-throughput efficiency with anatomical precision. While Vol2Brain enables rapid, atlas-based segmentation of global brain structures, it may lack the spatial granularity required to accurately delineate the complex foliated architecture of cerebellar lobules.

In contrast, semi-automated segmentation using 3D Slicer allows expert-guided refinement based on individual anatomical landmarks, such as the primary and horizontal fissures. This approach enhances the reliability of subregional cerebellar measurements, particularly in regions where fully automated boundary detection is prone to inaccuracies. To complement the automated cerebellar volumetric data obtained from Vol2Brain and to achieve a more detailed assessment of regional cerebellar morphology, an additional subregional analysis was therefore performed using 3D Slicer (version 5.6.1; Brigham and Women’s Hospital, Boston, MA, USA) [20].

3D Slicer is an open-source platform developed by Brigham and Women’s Hospital, the Massachusetts Institute of Technology (MIT), and the Laboratory for Surgical Navigation and Robotics. It enables semi-automated segmentation and volumetric measurement of high-resolution brain MRI data and is widely used for anatomically guided morphometric analyses [21]. This analysis was designed to extend beyond Vol2Brain’s fully automated atlas-based pipeline by enabling a more detailed morphometric assessment of the cerebellum at the lobular and vermian subregional levels. Based on prior evidence indicating that specific cerebellar subregions are functionally integrated within cognitive, emotional, and autonomic regulatory networks [22,23], a targeted cerebellar morphometric evaluation was incorporated into the present study. All images were retrieved as anonymized 3D T1-weighted MRI datasets from the institutional PACS archive. The DICOM data were imported into 3D Slicer, and semi-automated segmentation was performed using the Segment Editor module. Target structures were delineated through a combination of the Threshold, Region Growing, Paint, and Erase tools. For detailed cerebellar morphometry, a semi-automated segmentation protocol implemented in 3D Slicer was applied [20,21].

To ensure anatomical validity in the target pediatric age group and to minimize atlas-related misinterpretation, cerebellar boundaries were defined using subject-specific anatomical landmarks rather than relying solely on atlas-based registration. Characteristic cerebellar fissures—namely the fissura prima, fissura ansoparamediana, and fissura posterolateralis—were used as reliable anatomical markers, in accordance with the Schmahmann cerebellar atlas [24,25], as these fissures are well developed and consistently identifiable in pediatric MRI. Owing to the spatial resolution limitations of clinical MRI, cerebellar lobules I–II were evaluated as a single combined region. After segmentation, all cerebellar subregions were visually inspected in the axial, sagittal, and coronal planes to confirm anatomical accuracy. Three-dimensional surface models were generated using the Model Maker module, and volumetric measurements were automatically calculated with the Segment Statistics tool and normalized to total intracranial volume (ICV). The resulting measurement data were subsequently exported in .csv format for statistical analysis.

To ensure segmentation reliability, all regions of interest were manually reviewed by a neuroradiologist through inspection of corresponding X, Y, and Z coordinates, verifying precise alignment with the individual 3D T1-weighted images. In addition, a secondary quality control analysis was performed in a randomly selected subset comprising 10.2% of the study population. In this subset, manual segmentations performed by an experienced neuroradiologist were used to assess the consistency of the automated and semi-automated cerebellar parcellation.

Inter-rater reliability of the landmark-based 3D Slicer segmentation was evaluated using intraclass correlation coefficient (ICC) analysis, which demonstrated good to excellent reproducibility across the assessed cerebellar subregions. No systematic pipeline- or atlas-related missegmentations were identified during visual quality control. This semi-automated segmentation approach complemented the global volumetric outputs of Vol2Brain with regionally validated, anatomically detailed morphometric data, thereby yielding high-resolution and functionally relevant quantitative information on cerebellar substructures.

**Figure 1:**
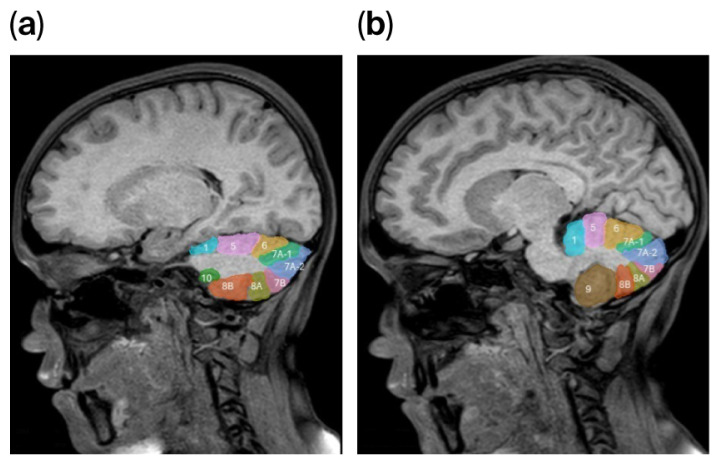
(**a**,**b**) Sagittal T1-weighted MR images showing the semi-automated segmentation of lobular anatomy in the right cerebellar hemisphere performed using 3D Slicer (version 5.6.1). Regions delineated with different color tones correspond to distinct lobular subregions defined according to Schmahmann’s cerebellar atlas. Numerical labels indicate individual cerebellar lobules, where lobule 7A-1 represents Crus I and lobule 7A-2 represents Crus II.

To illustrate the semi-automated segmentation process, Figure 1 presents the parcellation of the right cerebellar hemisphere as performed in 3D Slicer.

**Figure 2:**
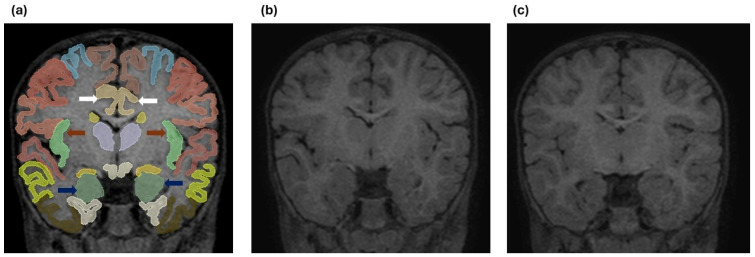
(**a**) Coronal T1-weighted MR image demonstrating automated cortical and subcortical parcellation performed using the Vol2Brain pipeline based on high-resolution 3D T1-weighted MRI data. Color-coded regions represent anatomically defined cortical and subcortical structures. Dark blue arrows indicate the bilateral amygdala, brown arrows indicate the bilateral anterior insular cortex, and white arrows indicate the bilateral caudal anterior cingulate gyrus. (**b**) Coronal 3D T1-weighted MR image obtained at a comparable anatomical level from a 1-year-old patient with breath-holding spells. (**c**) Coronal 3D T1-weighted MR image obtained at a similar level from a 1-year-old subject in the control group.

To illustrate the automated brain parcellation process, Figure 2 presents an example of cortical and subcortical segmentation performed using the Vol2Brain pipeline based on high-resolution 3D T1-weighted MRI data.

### 2.4. Statistical Analysis

All statistical analyses were performed using IBM SPSS Statistics (version 29.0; IBM Corp., Armonk, NY, USA) and R Studio (version 4.3.3; Posit Software, PBC, Boston, MA, USA). The distribution of continuous variables was assessed using the Shapiro–Wilk test, and variables that violated normality assumptions were log-transformed prior to parametric analyses when appropriate. Normally and non-normally distributed data are presented as mean ± standard deviation (SD) and median [interquartile range, IQR] values, respectively. Categorical variables are summarized as frequency (%).

Group differences in demographic and clinical characteristics were evaluated using the independent-samples *t*-test or Mann–Whitney U-test for continuous variables, and Fisher’s exact or the Chi-square test for categorical variables.

In the primary analysis, morphometric parameters (volume, surface area, cortical thickness) from the 135 anatomical regions derived by Vol2Brain were normalized to total intracranial volume (ICV) for each participant. Age and sex were included as covariates, and between-group comparisons were conducted using analysis of covariance (ANCOVA). To minimize the influence of developmental and physiological confounding, these factors—age, sex, and total intracranial volume—were treated as nuisance covariates in all volumetric comparisons. This approach ensures that the reported morphometric differences reflect group-level variations beyond those attributable to normative growth trajectories or individual head size. To account for potential non-linear developmental trajectories, we tested for quadratic effects of age (Age^2^); however, as these did not significantly improve model fit (*p* > 0.05) or alter the primary group-effect outcomes, age was retained as a linear covariate in the final models. To minimize false-positive findings arising from multiple regional comparisons, and because morphometric metrics are inter-correlated, the Benjamini–Hochberg false discovery rate (FDR) correction (q < 0.05) was applied instead of the overly conservative Bonferroni method. Two-tailed *p* < 0.05 was considered statistically significant.

Effect sizes (Hedges’ g) and 95% confidence intervals (CIs) were calculated for all significant regional differences. With the available sample (n = 98), the study had >80% power to detect medium effect sizes (g ≥ 0.5) at α = 0.05. To further evaluate the robustness of our findings, a sensitivity analysis was performed by sequentially excluding potential outliers (defined as values >3 SD from the mean); the results remained statistically consistent, indicating that the reported volumetric differences were not driven by individual extreme values.

In the secondary analysis, subregional cerebellar volumes obtained from 3D Slicer (v5.6.1) were evaluated using the same ANCOVA–FDR framework (q < 0.05).

Clinical correlation analyses were conducted as exploratory secondary tests specifically focusing on regions that demonstrated significant group differences. This approach was employed to identify potential clinical-structural relationships, recognizing the inherent selection step based on prior group-level significance. Brain regions showing significant group differences were further correlated with clinical parameters (spell frequency, duration, and subtype) within the BHS group, with correlations between continuous variables examined using Spearman’s rank correlation coefficient (ρ) and potential confounders controlled by performing partial correlations adjusted for age and sex. Significant associations were further verified using multivariate linear regression models. Negative correlation coefficients were interpreted as indicating that higher spell frequency was associated with lower regional volume or cortical thickness.

For variables with <20% missing data, median imputation was applied; those with >20% missing data were excluded using listwise deletion. Two-tailed *p* < 0.05 was considered statistically significant in all analyses. To ensure segmentation reliability, 10 randomly selected scans were re-segmented by the same researcher (intra-rater reliability) and by a second experienced neuroradiologist (inter-rater reliability). Intraclass correlation coefficient (ICC) analysis demonstrated good to excellent reproducibility for both intra- and inter-rater assessments, with values consistently falling within the 0.75–0.90 range, supporting the consistency of the semi-automated cerebellar segmentation approach.

## 3. Results

The most prominent finding of our analysis was the presence of selective, region-specific morphometric differences involving the amygdala, anterior insula, and specific cerebellar subregions—namely lobule VI, lobule VIIA, and vermis IX–X—in children with breath-holding spells compared with healthy controls. Regarding the study population, the BHS group included 25 boys (52.1%) and 23 girls (47.9%), while the control group comprised 26 boys (52.0%) and 24 girls (48.0%), meaning there was no significant difference in sex distribution between the two groups (*p* = 0.98). The mean age was 2.9 ± 1.4 years in the BHS group and 3.0 ± 1.2 years in the control group, again with no statistically significant difference (*p* = 0.67). The mean attack frequency in the BHS group was 2.8 ± 4.6 episodes per month with a right-skewed distribution, indicating that most children experienced infrequent attacks, whereas a small subset exhibited a high frequency (≥10 per month). The median attack duration was 1 min (IQR = 1–2), and 83.3% of patients experienced episodes lasting ≤2 min; only three children had episodes exceeding 5 min. Because of the non-parametric distribution of attack duration, median values were used for statistical comparisons.

Emotional stimuli—particularly crying episodes—were identified as the most common triggering factor (33.3%), followed by fear, sudden painful stimuli, and frustration. Regarding spell subtype distribution, 26 (54.2%) children presented with the cyanotic form, while 22 (45.8%) showed pallid (acyanotic) BHSs.

Morphometric analysis of 135 anatomical regions obtained through Vol2Brain, with age and sex included as covariates, revealed no statistically significant differences between groups in total intracranial volume, total brain volume, or gray or WM volume (all *p* > 0.05).

At the regional level, the BHS group demonstrated significantly reduced bilateral amygdala volumes (left: *p* = 0.011, q = 0.042; right: *p* = 0.007, q = 0.038), and the right anterior insular cortex showed significantly decreased GM thickness (*p* = 0.014, q = 0.046) and volume (*p* = 0.021, q = 0.049) compared with controls. Both anterior cingulate cortices (left: *p* = 0.019, q = 0.045; right: *p* = 0.017, q = 0.043) and the right medial frontal cortex (*p* = 0.009, q = 0.036) also demonstrated significantly reduced mean cortical thickness. No significant morphometric differences were observed in other anatomical regions after false discovery rate correction (all FDR-adjusted *p* > 0.05). Detailed statistical outcomes of these morphometric comparisons are presented in Table 1.

To complement the automated cerebellar volumetric data obtained from Vol2Brain and to enable a more detailed assessment of regional cerebellar morphology, an additional subregional analysis was performed using 3D Slicer (version 5.6.1; Brigham and Women’s Hospital, Boston, MA, USA). This analysis focused on voxel-based volumetric assessment at the lobular level. All volumetric values were normalized to each participant’s total intracranial volume (ICV), with age and sex included as covariates. Group differences were evaluated using analysis of covariance (ANCOVA), and the risk of type I error due to multiple comparisons was controlled with the Benjamini–Hochberg FDR correction (q < 0.05).

In the BHS group, a significant volume reduction was observed in the right lobule VI (*p* = 0.007, q = 0.031). Similarly, the left lobule VIIA [Crus I] volume was significantly lower than that in the control group (*p* = 0.012, q = 0.043). A marked volume decrease in the vermis IX–X region was detected (*p* = 0.010, q = 0.039). The right lobules VIIA [Crus II] (*p* = 0.046, q = 0.071) and VIIIB (*p* = 0.052, q = 0.078) showed trends toward significance but did not meet the predefined FDR-corrected threshold (q < 0.05). No other cerebellar subregions demonstrated significant volumetric differences (all *p* > 0.10). Detailed measurements and statistical outcomes are summarized in Table 2.

Correlations between clinical variables and regional brain morphometric measurements were examined within the BHS group. Brain regions that showed significant volumetric or cortical thickness differences in group comparisons (bilateral amygdala, right anterior insula, and right medial frontal cortex), along with cerebellar subregions exhibiting significant volumetric differences in 3D Slicer analysis (lobule VI and vermis IX–X), and cerebellar subregions showing a trend toward volumetric differences at the uncorrected level (lobules VIIA [Crus I–II] and VIIIB), were included in the correlation analysis.

A significant negative correlation was observed between attack frequency and the right amygdala volume (ρ = −0.41, *p* = 0.004, q = 0.021), right anterior insula volume (ρ = −0.37, *p* = 0.008, q = 0.034), and right medial frontal cortical thickness (ρ = −0.33, *p* = 0.012, q = 0.043). Among cerebellar regions, only the vermis IX–X volume showed a borderline significant negative correlation with attack frequency (ρ = −0.29, *p* = 0.037, q = 0.078).

No significant correlations were identified between attack duration and any regional volumetric or cortical thickness measurements (all *p* > 0.05), and similarly, spell type was not significantly associated with any volumetric parameters (all *p* > 0.05). The detailed correlation results are presented in Table 3.

## 4. Discussion

The key finding of this study is that BHSs, traditionally viewed as transient and functional episodes, may be associated with focal structural neurobiological variations within specific regulatory circuits of the developing brain. Our results suggest that the clinical manifestations of BHSs are linked to significant morphometric alterations within the central autonomic and limbic networks, specifically involving the amygdala–insula axis and specific cerebellar subregions. From a clinical perspective, these findings indicate that the vulnerability to respiratory and autonomic dysregulation in these children may not be merely physiological but could be related to the structural integrity of regions responsible for homeostatic control. To the best of our knowledge, this study represents the first quantitative neuroanatomical investigation to provide both global and subregional morphometric insights into the pediatric BHS population, moving the understanding of this condition beyond simple paroxysmal events toward a model of potential structural susceptibility in the brain’s emotional and autonomic regulatory circuits. The cerebellum is not solely a motor coordination center but also an integral component of the emotional processing, autonomic regulation, and cognitive control networks [22,23]. In particular, the vermis and posterior lobules (VI–X) form bidirectional connections with the hypothalamus and limbic system, playing a pivotal role in modulating cardiac rhythm, vasomotor responses, and emotional arousal [24,25]. The frontal lobe, especially the medial and anterior cingulate cortices, is a key cortical hub responsible for behavioral inhibition, emotional regulation, and autonomic response integration [3]. In the present study, volume reductions in the amygdala, anterior insula, medial frontal cortex, and selected cerebellar subregions—specific nodes within these regulatory circuits—were observed, with several showing significant negative correlations with attack frequency. In addition, reduced cortical thickness of the anterior cingulate cortex in the BHS group provides structural support for its involvement in the limbic–frontal alterations observed in this condition, consistent with its established role in emotional–autonomic integration [3,4]. In contrast, attack duration and spell type were not significantly associated with any morphometric measure. Although the observed morphometric differences involved key regions implicated in autonomic and emotional regulation, including the bilateral amygdala, right anterior insula, anterior cingulate cortex, and medial frontal cortex, they did not form a fully coherent or symmetric anatomical pattern. This spatial heterogeneity may be consistent with the known functional asymmetry of the autonomic nervous system, where the right hemisphere often plays a more prominent role in sympathetic arousal [3]. Furthermore, these findings may reflect the exploratory design of the study, interindividual variability in early neurodevelopment, and limitations related to sample size. Accordingly, the present findings should be interpreted as hypothesis-generating rather than as evidence of a definitive neuroanatomical network. These findings suggest that the developing brain may exhibit localized volumetric adaptations in response to transient hemodynamic shifts. Such effects likely represent subtle structural signatures within the limbic, frontal, and cerebellar networks, reflecting the brain’s early response to recurrent paroxysmal events [26,27,28]. These findings potentially carry clinical implications when considered within the context of typical pediatric neurodevelopmental trajectories. During early childhood, the cerebellum is known to undergo rapid structural maturation, which is thought to be critical for the development of motor coordination and autonomic stability [22,23]. The lower volumes observed in our cohort might reflect a subtle developmental variance or a transient maturational lag relative to established pediatric norms [2,24]. Clinically, such neuroanatomical observations could be explored as a possible structural correlate for the autonomic dysregulation seen in BHSs. This suggests that the condition may warrant further investigation into subclinical structural variations in homeostatic control centers, rather than being viewed as a strictly functional phenomenon [3,9].

Brain development during early childhood is characterized by rapid and nonlinear maturational processes, including region-specific changes in gray and white matter volumes [6,7,8]. To account for developmental effects, age was incorporated as a covariate in all morphometric analyses. Although age-related subgroup analyses or nonlinear modeling approaches may provide additional developmental resolution, such stratification was not performed in the present cohort, as subdividing the relatively broad pediatric age range into multiple age groups would substantially increase the number of statistical comparisons, reduce subgroup sample sizes, and elevate the risk of type I error. Accordingly, volumetric findings were interpreted within the context of ongoing rapid neurodevelopmental maturation, while emphasizing associations with clinical variables rather than age-specific maturational effects.

Neuroimaging studies on BHSs remain extremely limited, and no prior research has directly addressed structural morphometry. For example, Ozcora et al. [2] investigated brainstem microstructure in children with severe BHSs using diffusion tensor imaging (DTI) but found no significant differences in fractional anisotropy or mean diffusivity values. Similarly, Calik et al. [26] analyzed global metabolic profiles with proton magnetic resonance spectroscopy (^1^H-MRS) and reported no significant group differences in NAA/Cr, Cho/Cr, or mI/Cr ratios. These findings highlight the lack of evidence for macroscopic or microscopic brain alterations in BHSs, suggesting that potential morphometric effects have remained unexplored. In line with this, Leung et al. [1], in a recent review, noted the absence of morphometric, volumetric, or voxel-based studies, emphasizing that prior research has predominantly focused on autonomic regulation and neurophysiological mechanisms rather than structural imaging.

The present study’s findings indicate that BHSs in childhood may not be limited to transient autonomic episodes but, in certain cases, may be associated with measurable structural variations in neurodevelopmentally sensitive brain regions. Morphometric alterations observed in limbic, frontal, and cerebellar areas raise the possibility that recurrent spells could exert subtle but non-negligible neurodevelopmental effects. Therefore, structural brain assessment could serve not only as a diagnostic adjunct but also as a supportive tool for neurodevelopmental monitoring in children with a history of BHSs [27,28]. The quantitative volumetric methods employed in this study provide objective neuroanatomical documentation that may inform and guide future large-scale longitudinal studies.

Multiple mechanisms may underpin these morphometric variations. Specifically, recurrent episodes of hypoxia and reperfusion may trigger metabolic stress, neurovascular reactivity alterations, and synaptic remodeling. Given the high metabolic demand of the amygdala and cerebellum during early childhood, these regions may be particularly susceptible to the physiological stress induced by transient hypoperfusion, leading to the subtle volumetric differences observed in our cohort [28]. These mechanisms could result in subtle volumetric adaptations over time, particularly within limbic and cerebellar structures. However, the detected morphometric variations might also represent secondary or compensatory changes rather than direct pathophysiological effects. Additionally, individual anatomical variability, genetic predisposition, and differences in developmental maturation rates could influence volumetric outcomes [28]. Given that BHSs typically occur in early childhood—a period characterized by rapid brain maturation and intense synaptic organization—physiological developmental variability must also be considered. Thus, the observed volumetric alterations may reflect both spell-related and developmental influences. These findings collectively underscore the need for more detailed, region-specific analyses in order to elucidate the extent and anatomical specificity of potential structural sensitivity in early BHSs [27,28]. It remains unclear whether the observed morphometric variations represent pre-existing neuroanatomical characteristics that may predispose individuals to BHSs or whether they emerge as secondary or compensatory changes related to recurrent episodes, developmental variability, genetic predisposition, or other unmeasured environmental factors. While the involvement of the amygdala, insula, and specific cerebellar subregions points toward potential structural targets in BHSs, the absence of alterations in other functionally contiguous regions—such as the orbitofrontal cortex or brainstem—and the lack of anatomical symmetry suggest that these findings do not yet constitute a complete or universal neurobiological network. These results should be viewed as localized regions of susceptibility rather than a comprehensive system-wide deficit. However, given the cross-sectional nature of the study and the focal rather than global pattern of differences, these findings are best interpreted as indicative of potential neuroanatomical susceptibility within autonomic regulatory circuits rather than evidence of cumulative brain injury.

Vol2Brain software (version 1.0, release 23 November 2021) provides a major methodological advantage by enabling whole-brain volumetric assessment with high automation and reproducibility [11]. This approach aligns with the broader evolution of neuroimaging, where unified algorithms for multimodal MRI and the integration of machine learning—highly successful in complex tasks such as glioma and brain tumor segmentation have significantly improved the precision of structural analysis [29]. However, its atlas-based segmentation approach offers limited resolution for small, morphologically complex structures such as cerebellar sublobules. Considering the cerebellum’s unique neurodevelopmental role and the biological plausibility of its involvement in BHSs, a more detailed subregional analysis was warranted. Therefore, semi-automated cerebellar segmentation using 3D Slicer [20,21] was employed to complement the Vol2Brain results at the lobular level. This approach allowed for the detection of subtle volume differences with greater sensitivity, enhancing the reliability of findings in functionally critical regions such as the vermis IX–X. Integrating these high-resolution cerebellar data with global morphometric measures enabled a more anatomically precise assessment of the structural impact of BHSs.

This study has several limitations. First, the most significant methodological limitation is its cross-sectional design, which precludes any definitive conclusions regarding temporality or reverse causality. Without longitudinal data following patients from the pre-symptomatic stage through the resolution of the spells, it remains impossible to definitively differentiate between brain maturation effects influenced by BHSs and pre-existing neuroanatomical substrates that may have preceded the clinical onset of the disorder. Second, its single-center, retrospective design limits sample diversity and generalizability. Third, beyond the primary covariates controlled in our model, other unmeasured developmental factors—such as gestational age, birth weight, nutritional status, socioeconomic background, and specific metabolic parameters (e.g., serum iron, ferritin, and vitamin B12)—were not consistently available across the cohort. Given that these factors are known to influence pediatric brain morphometry, they represent a potential source of residual confounding that should be considered when interpreting the specificity of our findings. Fourth, BHSs comprise clinically distinct cyanotic and pallid subtypes, which may differ in underlying autonomic mechanisms. Although both subtypes were represented in the present cohort, the study was not designed or powered to perform reliable subtype-specific morphometric analyses. Therefore, the potential heterogeneity between BHS subtypes should be considered when interpreting the results, and future studies with larger, subtype-stratified cohorts may help clarify whether distinct neuroanatomical patterns exist. Fifth, our clinical correlation analyses were restricted to brain regions that first demonstrated significant group differences. While this targeted approach helps link structural findings to clinical severity, it may involve a degree of selection bias (circular analysis) that could potentially overstate the strength of these associations. Consequently, these findings should be interpreted as exploratory evidence of clinical relevance rather than independent validation. Sixth, a significant limitation concerns the recruitment of the control group; although all control subjects had structurally normal brain MRI scans and normal neurological examinations, they were recruited from a clinical population (e.g., referred for headache or dizziness) rather than a truly asymptomatic general population. Therefore, the control group may not fully represent the general pediatric population, and subtle neurobiological differences unrelated to BHSs cannot be entirely excluded. This selection bias should be considered when interpreting group-level morphometric differences. Seventh, although all scans were performed without sedation, ensuring the absence of pharmacological interference, the reliance on spontaneous natural sleep may have introduced mild motion artifacts in some cases. While we excluded scans with significant artifacts to maintain the reliability of the morphometric data, this methodological choice should be considered when evaluating the final cohort. Eighth, our interpretation regarding the role of intermittent hypoxia and hypoperfusion is inherently limited by the lack of direct physiological monitoring (e.g., oxygen saturation or cerebral blood flow measurements) during the spells. While breath-holding episodes are typically brief, the cumulative neurobiological impact of recurrent paroxysmal events on the developing brain remains a subject of ongoing investigation. Consequently, the proposed link between these episodes and structural variations should be regarded as a plausible mechanistic framework rather than a definitive causal relationship. Finally, because all participants were in early childhood, ongoing neurodevelopmental maturation may have influenced morphometric variability.

Despite these limitations, the present study offers novel, quantitative evidence suggesting that recurrent BHSs might be associated with subtle structural signatures in the developing brain. By establishing a robust methodological framework that integrates global automated pipelines (Vol2Brain) with high-resolution subregional cerebellar mapping (3D Slicer), this work represents the first investigation in the BHS literature to characterize a distinct topographic pattern of morphometric variations across limbic, frontal, and cerebellar networks. These findings suggest that the amygdala, anterior insula, and medial frontal cortex—regions essential for emotional-autonomic regulation—may provide a potential anatomical framework for the dysregulation characteristic of BHSs. Furthermore, the identified associations between these structural variations and clinical severity might indicate that transient hypoperfusion episodes could be linked to subtle adaptations in neurodevelopmentally sensitive areas. Although the identification of region-specific morphometric differences may provide insights into the neurobiological substrates of BHSs, the magnitude and variability of these findings do not support their use as diagnostic or prognostic markers at the individual level. Accordingly, structural MRI findings in this context should be interpreted as research-level observations that may inform future hypothesis-driven and longitudinal investigations, rather than as tools for immediate clinical application. Ultimately, these preliminary observations warrant further investigation through prospective, multicenter, and longitudinal studies to determine the precise nature of these developmental dynamics.

## 5. Conclusions

This study represents the first comprehensive morphometric evaluation of children with BHSs, integrating multiple segmentation tools and analysis methods. Our findings demonstrate significant volumetric differences, particularly within the limbic, frontal, and cerebellar regions. Given the retrospective and cross-sectional nature of this research, these results should be interpreted as associations rather than definitive causal effects. Nevertheless, the data suggests that breath-holding spells may be linked to subtle neurodevelopmental variability during early childhood, rather than being strictly transient autonomic events. Future longitudinal studies that track age-related structural changes alongside neuropsychological assessments will be essential to clarify the clinical significance and long-term trajectory of these findings.

## Figures and Tables

**Table 1:** Group comparisons of morphometric parameters derived from automated volumetric analysis (Vol2Brain) adjusted for age and sex using ANCOVA.

Anatomical Region	Measurement Type	Breath-Holding Spells (n = 48) Mean ± SD	Control (n = 50) Mean ± SD	*p*-Value	q (FDR)	Effect Size (Hedges g) [95% CI]
Amygdala (L)	Volume (mm^3^)	1263 ± 172	1401 ± 183	**0.011**	**0.042**	0.52 [0.12, 0.92]
Amygdala (R)	Volume (mm^3^)	1277 ± 168	1436 ± 191	**0.007**	**0.038**	0.56 [0.16, 0.96]
Anterior Insula (R)	Cortical Thickness (mm)	2.59 ± 0.21	2.73 ± 0.19	**0.014**	**0.046**	0.49 [0.09, 0.89]
Anterior Insula (R)	Volume (mm^3^)	5728 ± 554	6012 ± 523	**0.021**	**0.049**	0.41 [0.02, 0.80]
Anterior Cingulate Cortex (L)	Cortical Thickness (mm)	2.49 ± 0.20	2.61 ± 0.18	**0.019**	**0.045**	0.43 [0.04, 0.82]
Anterior Cingulate Cortex (R)	Cortical Thickness (mm)	2.51 ± 0.22	2.65 ± 0.19	**0.017**	**0.043**	0.45 [0.06, 0.84]
Medial Frontal Cortex (R)	Cortical Thickness (mm)	2.61 ± 0.18	2.74 ± 0.17	**0.009**	**0.036**	0.52 [0.12, 0.91]
Remaining 128 regions	—	—	—	N.S.	—	—

All values are adjusted for age and sex using ANCOVA. *p* < 0.05 indicates statistical significance; q < 0.05 indicates significance after Benjamini–Hochberg FDR correction. Volumes are normalized to total intracranial volume (ICV). Significant results are shown in bold.

**Table 2:** Comparison of cerebellar subregional volumes obtained by semi-automated segmentation in 3D Slicer (ICV-normalized, age- and sex-adjusted ANCOVA).

Anatomical Region	Side	BHS Mean ± SD	Control Mean ± SD	*p*	q (FDR)	Effect Size Hedges g [95% CI]
Lobule I–II	Right	2345 ± 271	2362 ± 267	0.68	0.82	0.06 [−0.33, 0.45]
Lobule I–II	Left	2317 ± 259	2332 ± 262	0.74	0.85	0.05 [−0.34, 0.44]
Lobule III	Right	1802 ± 206	1826 ± 201	0.48	0.71	0.12 [−0.27, 0.51]
Lobule III	Left	1783 ± 197	1804 ± 205	0.54	0.73	0.10 [−0.29, 0.49]
Lobule IV–V	Right	3944 ± 375	4015 ± 384	0.39	0.67	0.16 [−0.23, 0.55]
Lobule IV–V	Left	3911 ± 366	3979 ± 379	0.42	0.69	0.15 [−0.24, 0.54]
Lobule VI	Right	4556 ± 447	4869 ± 463	**0.007**	**0.031**	0.50 [0.10, 0.89]
Lobule VI	Left	4501 ± 442	4629 ± 451	0.19	0.46	0.21 [−0.18, 0.60]
Lobule VIIA (Crus I)	Left	3934 ± 392	4213 ± 399	**0.012**	**0.043**	0.44 [0.05, 0.83]
Lobule VIIA (Crus I)	Right	3982 ± 404	4087 ± 411	0.064	0.089	0.29 [−0.10, 0.68]
Lobule VIIA (Crus II)	Right	2731 ± 326	2806 ± 318	0.046	0.071	0.33 [−0.06, 0.72]
Lobule VIIA (Crus II)	Left	2719 ± 317	2758 ± 325	0.28	0.59	0.17 [−0.22, 0.56]
Lobule VIIIA	Right	2193 ± 263	2237 ± 270	0.35	0.63	0.14 [−0.25, 0.53]
Lobule VIIIA	Left	2185 ± 258	2226 ± 266	0.38	0.66	0.13 [−0.26, 0.52]
Lobule VIIIB	Right	1654 ± 188	1693 ± 190	0.052	0.078	0.31 [−0.08, 0.70]
Lobule VIIIB	Left	1649 ± 183	1678 ± 189	0.41	0.69	0.15 [−0.24, 0.54]
Vermis VIII	Midline	1336 ± 142	1387 ± 149	0.083	0.117	0.26 [−0.13, 0.65]
Vermis IX–X	Midline	1121 ± 128	1247 ± 139	**0.010**	**0.039**	0.47 [0.07, 0.86]
Lobule IX	Right	1977 ± 228	1998 ± 233	0.53	0.72	0.09 [−0.30, 0.48]
Lobule IX	Left	1962 ± 226	1982 ± 228	0.57	0.74	0.08 [−0.31, 0.47]
Lobule X	Right	951 ± 118	966 ± 120	0.44	0.69	0.12 [−0.27, 0.51]
Lobule X	Left	944 ± 116	958 ± 119	0.46	0.71	0.11 [−0.28, 0.50]

All volumes are normalized to total intracranial volume (ICV). Analyses were adjusted for age and sex using ANCOVA with Benjamini–Hochberg FDR correction (q < 0.05). Values in **bold** are statistically significant. 95% CI: 95% Confidence Interval for Hedges’ g. Intra- and inter-rater ICC analysis demonstrated good-to-excellent segmentation reliability (ICC range: 0.75–0.90).

**Table 3:** Correlations between clinical parameters and regional morphometric measures in children with breath-holding spells (Spearman ρ).

Anatomical Region	Spell Frequency (ρ)	*p*	q (FDR)	Spell Duration (ρ)	*p*	q (FDR)	Spell Type *p*
Amygdala (R)	−0.41	0.004	0.021	−0.12	0.362	0.603	0.48
Amygdala (L)	−0.24	0.074	0.181	−0.10	0.405	0.628	0.57
Anterior Insula (R)	−0.37	0.008	0.034	−0.09	0.423	0.637	0.52
Medial Frontal Cortex (R)	−0.33	0.012	0.043	−0.15	0.285	0.581	0.60
Vermis IX–X	−0.29	0.037	0.078	−0.11	0.377	0.619	0.63
Lobule VI (R)	−0.26	0.081	0.161	−0.08	0.465	0.654	0.59
Lobule VIIA (Crus I-L)	−0.25	0.094	0.177	−0.14	0.308	0.598	0.68
Lobule VIIA (Crus II-R)	−0.18	0.186	0.283	−0.05	0.577	0.697	0.70
Lobule VIIIB (R)	−0.20	0.154	0.254	−0.07	0.496	0.668	0.66

n = 48 (BHS group only). Correlations were computed using Spearman’s rank test and corrected for multiple comparisons by Benjamini–Hochberg FDR (q < 0.05). Negative correlation coefficients indicate that greater spell frequency is associated with reduced regional volume or cortical thickness. “Spell Type” refers to cyanotic versus pallid subtype.

## Data Availability

The MRI datasets generated and analyzed during the current study are not publicly available due to institutional data protection policies but are available from the corresponding author on reasonable request.

## Supplementary Materials

The following supporting information can be downloaded at: https://www.mdpi.com/article/10.3390/tomography12020021/s1; Supplementary File S1: Vol2Brain volumetry report.
