# Supplementary material for: Comprehensive Morphometric MRI Assessment in Children with Breath-Holding Spells: Integration of Automated (Vol2Brain) and Semi-Automated (3D Slicer) Segmentation Methods
Source: Tomography. 2026 Feb 6;12(2):21. doi: 10.3390/tomography12020021 (PMC12944239; doi:10.3390/tomography12020021)
Supplement: Supplementary file 1 [file tomography-12-00021-s001.zip › tomography-4091859-supplementary.pdf]

vol2Brain volumetry report

version 1.0 release 23-11-2021.

Subject: job1912061

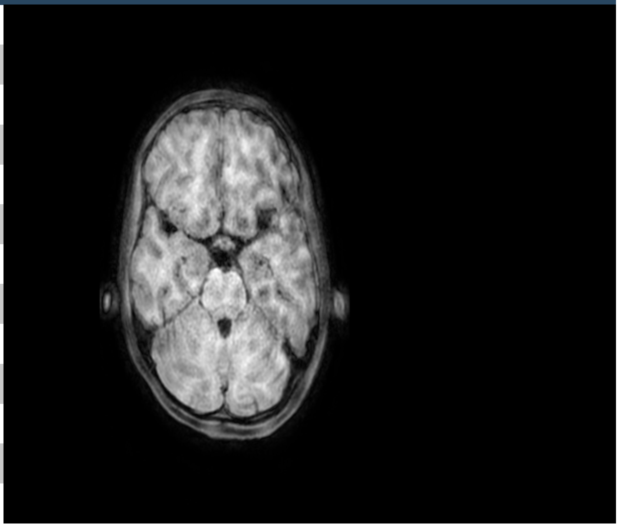

**Sex**	Male
**Age**	6
**Report date**	02-Nov-2025
**Image orientation**	neurological
**Scale factor**	0.80
**SNR**	67.89

Tissue segmentation

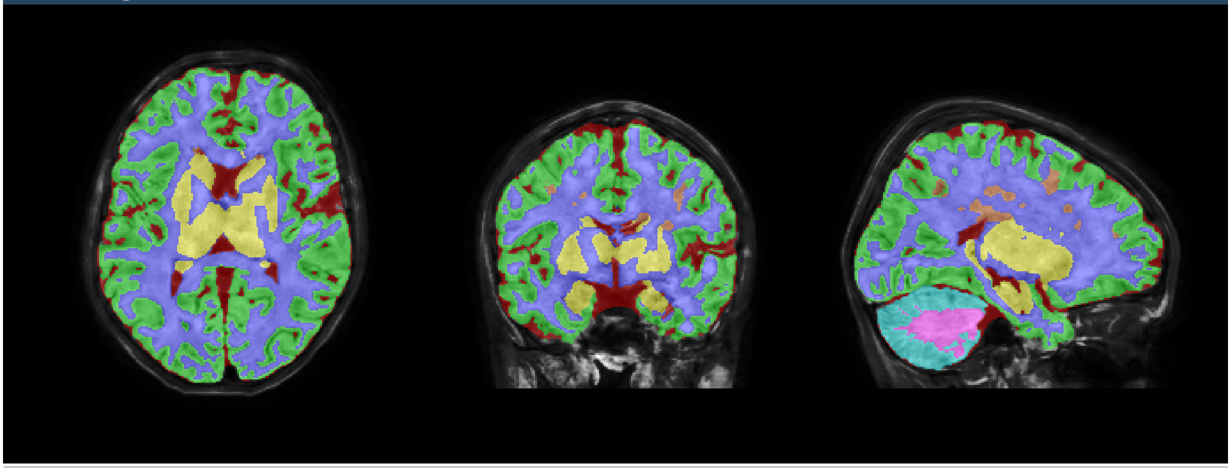

Tissue	Volume (cm^3/%)	
**White Matter (WM)**	502.70 (35.692)	[29.757, 39.591]
Normal Appearing White Matter	494.14 (35.085)	[29.636, 39.480]
Abnormal Appearing White Matter	8.56 (0.608)	[0.000, 0.321]
**Grey Matter (GM)**	732.14 (51.983)	[50.580, 58.498]
Subcortical Grey Matter	44.02 (3.126)	[2.864, 3.586]
Cortical Grey Matter	592.77 (42.088)	[39.613, 46.471]
Cerebellar Grey Matter	95.35 (6.770)	[7.072, 9.472]
**Cerebro Spinal Fluid (CSF)**	154.67 (10.982)	[4.688, 14.144]
**Brain (WM+GM)**	1234.84 (87.675)	[84.527, 93.899]
**Intracranial Cavity (IC)**	1408.42 (100.000)	[100.000, 100.000]

**All the volumes are presented in absolute value (measured in cm^3) and in relative value (measured in relation to the ICV).*

**The Asymmetry Index is calculated as the difference between right and left volumes divided by their mean (in percent).*

**Segmentation images are located in the MNI space (neurological orientation).*

**Values between brackets show expected limits (95%) of normalized volume in function of sex and age for each measure for reference purpose. Values outside the limits are highlighted in red.*

Macrostructures

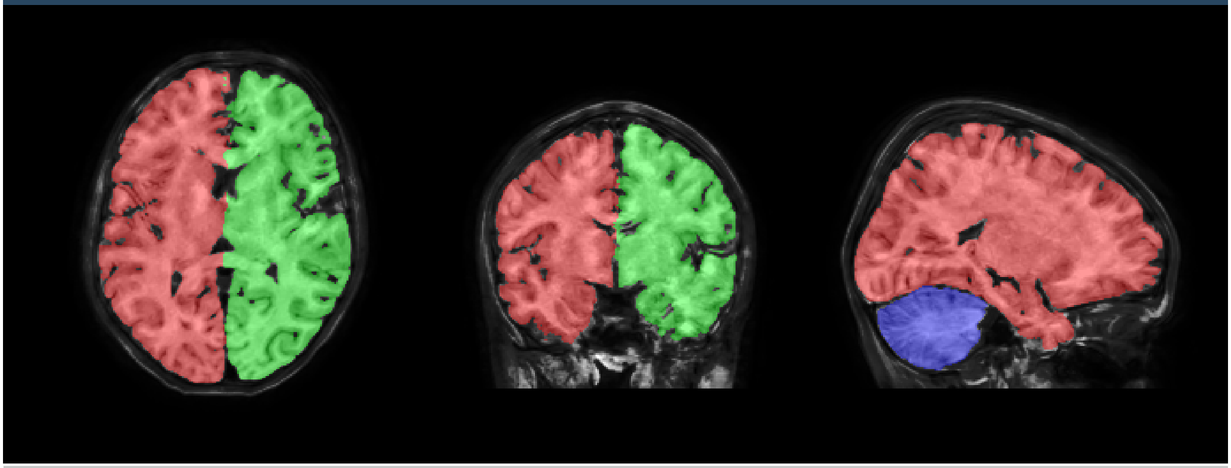

Structure	Total (cm^3/%)	Right (cm^3/%)	Left (cm^3/%)	Asymmetry (%)
**Cerebrum**	1110.16 (78.823)<br>[74.128, 83.501]	556.31 (39.499)<br>[36.911, 41.708]	553.85 (39.324)<br>[37.172, 41.838]	0.4422<br>[-2.228, 1.215]
Cerebrum WM	473.36 (33.609)<br>[27.876, 37.219]	243.67 (17.301)<br>[13.862, 18.655]	229.70 (16.309)<br>[13.996, 18.582]	5.9029<br>[-2.599, 2.266]
Cerebrum GM	636.79 (45.213)<br>[42.699, 49.834]	312.64 (22.198)<br>[21.277, 24.825]	324.15 (23.015)<br>[21.408, 25.024]	-3.6169<br>[-2.424, 0.801]
**Cerebellum ***	115.87 (8.227)<br>[8.364, 11.006]	58.49 (4.153)<br>[4.178, 5.533]	57.38 (4.074)<br>[4.176, 5.484]	1.9095<br>[-3.164, 4.201]
Cerebellum WM	29.33 (2.083)<br>[1.607, 2.645]	15.76 (1.119)<br>[0.797, 1.331]	13.57 (0.964)<br>[0.806, 1.319]	14.9542<br>[-5.700, 6.333]
Cerebellum GM	95.35 (6.770)<br>[7.072, 9.472]	42.73 (3.034)<br>[3.218, 4.364]	43.81 (3.111)<br>[3.219, 4.317]	-2.5122<br>[-3.651, 5.041]
**Vermis**	8.81 (0.625)<br>[0.578, 0.849]			
**Brainstem**	18.91 (1.343)<br>[1.159, 1.583]			

**Cerebellum volumes does not include vermis volume.*

Structure segmentation

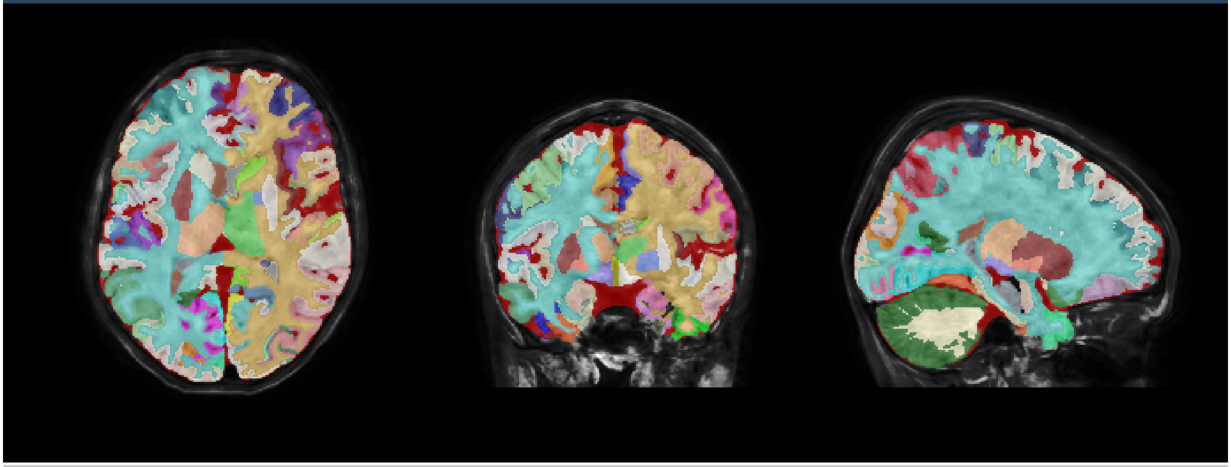

Subcortical	Total (cm^3/%)	Right (cm^3/%)	Left (cm^3/%)	Asymmetry (%)
Accumbens	0.65 (0.047)<br>[0.036, 0.068]	0.30 (0.021)<br>[0.016, 0.033]	0.36 (0.025)<br>[0.019, 0.036]	-17.6904<br>[-40.623, 20.252]
Amygdala	1.63 (0.115)<br>[0.106, 0.167]	0.75 (0.053)<br>[0.052, 0.083]	0.87 (0.062)<br>[0.052, 0.085]	-15.1410<br>[-16.777, 13.962]
Basal Forebrain	0.61 (0.044)<br>[0.039, 0.071]	0.35 (0.025)<br>[0.019, 0.037]	0.27 (0.019)<br>[0.018, 0.037]	24.6073<br>[-39.552, 32.222]
Caudate	7.34 (0.521)<br>[0.484, 0.717]	3.08 (0.218)<br>[0.241, 0.357]	4.26 (0.303)<br>[0.241, 0.362]	-32.3684<br>[-9.943, 7.282]
Hippocampus	7.42 (0.527)<br>[0.416, 0.625]	3.99 (0.283)<br>[0.202, 0.314]	3.43 (0.243)<br>[0.210, 0.316]	15.1177<br>[-13.574, 9.591]
Pallidum	2.81 (0.200)<br>[0.181, 0.269]	1.81 (0.129)<br>[0.089, 0.135]	1.00 (0.071)<br>[0.090, 0.136]	57.2899<br>[-14.386, 9.978]
Putamen	9.54 (0.678)<br>[0.608, 0.852]	4.72 (0.335)<br>[0.301, 0.422]	4.82 (0.342)<br>[0.305, 0.433]	-2.0570<br>[-10.408, 6.495]
Thalamus	14.01 (0.995)<br>[0.778, 1.032]	6.57 (0.467)<br>[0.384, 0.510]	7.44 (0.528)<br>[0.391, 0.525]	-12.3255<br>[-9.937, 4.280]
Ventral DC	8.92 (0.634)<br>[0.643, 0.824]	4.38 (0.311)<br>[0.313, 0.406]	4.54 (0.322)<br>[0.328, 0.420]	-3.5347<br>[-8.841, 1.026]

Cortical	Total (cm^3/%)	Right (cm^3/%)	Left (cm^3/%)	Asymmetry (%)
**Frontal lobe**	200.28 (14.220) [13.419, 16.078]	100.34 (7.125) [6.665, 8.042]	99.93 (7.095) [6.720, 8.069]	0.4114 [-5.221, 4.381]
Frontal pole	6.99 (0.496) [0.398, 0.652]	3.19 (0.227) [0.196, 0.366]	3.80 (0.269) [0.193, 0.340]	-17.3232 [-30.156, 21.711]
Gyrus rectus	3.78 (0.269) [0.222, 0.372]	1.88 (0.134) [0.109, 0.198]	1.90 (0.135) [0.102, 0.184]	-1.0636 [-25.982, 37.930]
Opercular inf. frontal gyrus	8.05 (0.572) [0.430, 0.688]	4.23 (0.300) [0.184, 0.347]	3.82 (0.271) [0.212, 0.376]	10.2129 [-55.747, 30.904]
Orbital inf. frontal gyrus	3.68 (0.261) [0.159, 0.342]	1.59 (0.113) [0.083, 0.192]	2.09 (0.148) [0.049, 0.176]	-27.1972 [-57.260, 106.2]
Triangular inf. frontal gyrus	7.66 (0.544) [0.451, 0.711]	3.75 (0.266) [0.210, 0.382]	3.91 (0.278) [0.204, 0.365]	-4.2875 [-38.596, 44.743]
Medial frontal cortex	3.80 (0.270) [0.202, 0.343]	1.85 (0.131) [0.092, 0.190]	1.95 (0.138) [0.088, 0.175]	-5.3825 [-42.287, 63.460]
Middle frontal gyrus	47.15 (3.348) [2.954, 3.780]	25.66 (1.822) [1.458, 1.925]	21.50 (1.526) [1.457, 1.894]	17.6600 [-12.270, 13.805]
Anterior orbital gyrus	4.22 (0.300) [0.234, 0.394]	1.83 (0.130) [0.113, 0.211]	2.39 (0.170) [0.103, 0.200]	-26.8395 [-38.387, 45.959]
Lateral orbital gyrus	4.26 (0.303) [0.246, 0.456]	2.34 (0.166) [0.117, 0.239]	1.92 (0.136) [0.110, 0.236]	19.6981 [-42.831, 49.244]
Medial orbital gyrus	7.37 (0.523) [0.305, 0.601]	3.40 (0.241) [0.236, 0.361]	3.97 (0.282) [0.136, 0.308]	-15.6298 [-25.681, 17.688]
Posterior orbital gyrus	5.88 (0.418) [0.369, 0.598]	2.10 (0.149) [0.161, 0.296]	3.78 (0.268) [0.192, 0.319]	-56.9435 [-42.085, 20.981]
Precentral gyrus	30.37 (2.157) [1.809, 2.384]	16.28 (1.156) [0.886, 1.219]	14.09 (1.001) [0.888, 1.200]	14.4106 [-15.191, 17.513]
Precentral gyrus medial segment	6.54 (0.464) [0.318, 0.491]	3.34 (0.237) [0.158, 0.265]	3.20 (0.227) [0.139, 0.247]	4.4040 [-23.907, 41.057]
Subcallosal area	2.92 (0.208) [0.024, 0.209]	1.29 (0.092) [0.013, 0.112]	1.63 (0.116) [0.007, 0.101]	-23.6039 [-15.245, 31.046]
Sup. frontal gyrus	29.73 (2.111) [2.162, 2.883]	13.91 (0.988) [1.073, 1.462]	15.82 (1.123) [1.052, 1.458]	-12.8021 [-14.729, 18.134]
Sup. frontal gyrus medial segment	15.62 (1.109) [0.919, 1.298]	7.93 (0.563) [0.421, 0.671]	7.69 (0.546) [0.444, 0.680]	3.0088 [-39.335, 27.851]
Supplementary motor cortex	12.25 (0.869) [0.661, 0.978]	5.78 (0.410) [0.316, 0.502]	6.47 (0.459) [0.311, 0.510]	-11.3272 [-30.359, 30.167]

**Temporal lobe**	116.42 (8.266) [7.251, 9.008]	57.17 (4.059) [3.630, 4.539]	59.25 (4.207) [3.585, 4.503]	-3.5676 [-6.036, 8.371]
Fusiform gyrus	15.68 (1.113) [0.970, 1.468]	6.48 (0.460) [0.477, 0.762]	9.20 (0.653) [0.462, 0.737]	-34.6746 [-47.683, 12.562]
Planum polare	3.86 (0.274) [0.259, 0.382]	1.81 (0.129) [0.119, 0.193]	2.05 (0.145) [0.127, 0.202]	-12.0492 [-35.988, 24.348]
Planum temporale	4.36 (0.309) [0.246, 0.426]	2.04 (0.145) [0.120, 0.224]	2.31 (0.164) [0.105, 0.223]	-12.2992 [-35.415, 55.207]
Inf. temporal gyrus	22.84 (1.622) [1.454, 2.105]	11.34 (0.805) [0.680, 1.040]	11.50 (0.816) [0.729, 1.110]	-1.3669 [-28.577, 14.301]
Middle temporal gyrus	33.19 (2.357) [2.109, 2.746]	17.03 (1.209) [1.010, 1.376]	16.16 (1.148) [1.048, 1.421]	5.2162 [-23.206, 13.959]
Sup. temporal gyrus	16.94 (1.203) [1.059, 1.599]	8.79 (0.624) [0.575, 0.897]	8.16 (0.579) [0.427, 0.758]	7.4651 [-18.373, 36.174]
Transverse temporal gyrus	3.61 (0.256) [0.162, 0.318]	1.73 (0.123) [0.068, 0.154]	1.88 (0.133) [0.082, 0.177]	-8.2999 [-57.219, 24.636]
Temporal pole	15.94 (1.132) [1.137, 1.747]	7.95 (0.564) [0.600, 0.906]	8.00 (0.568) [0.515, 0.863]	-0.6359 [-8.291, 28.841]
**Parietal lobe**	118.76 (8.432) [8.019, 9.853]	59.30 (4.211) [3.985, 4.932]	59.45 (4.221) [3.995, 4.960]	-0.2520 [-7.246, 6.402]
Angular gyrus	25.39 (1.803) [1.369, 2.111]	13.71 (0.973) [0.693, 1.103]	11.68 (0.829) [0.643, 1.041]	15.9579 [-17.020, 29.888]
Postcentral gyrus	22.09 (1.569) [1.231, 1.714]	10.75 (0.763) [0.590, 0.879]	11.34 (0.805) [0.601, 0.875]	-5.3755 [-21.588, 20.635]
Postcentral gyrus medial segment	1.73 (0.123) [0.081, 0.173]	0.84 (0.060) [0.036, 0.094]	0.89 (0.063) [0.032, 0.091]	-5.1092 [-56.919, 62.804]
Precuneus	25.26 (1.793) [1.479, 2.052]	12.26 (0.870) [0.718, 1.037]	13.00 (0.923) [0.738, 1.038]	-5.9058 [-16.108, 13.424]
Sup. parietal lobule	24.30 (1.725) [1.249, 1.777]	11.84 (0.840) [0.585, 0.888]	12.46 (0.885) [0.627, 0.927]	-5.1462 [-26.152, 16.287]
Supramarginal gyrus	19.98 (1.419) [1.282, 1.740]	9.91 (0.704) [0.600, 0.865]	10.08 (0.715) [0.640, 0.917]	-1.6669 [-33.088, 17.769]

**Occipital lobe**	78.54 (5.576) [4.608, 6.193]	38.81 (2.755) [2.315, 3.175]	39.73 (2.821) [2.223, 3.088]	-2.3522 [-9.583, 16.737]
Calcarine cortex	6.18 (0.439) [0.279, 0.609]	2.48 (0.176) [0.139, 0.314]	3.70 (0.263) [0.129, 0.307]	-39.4583 [-24.645, 34.007]
Cuneus	8.41 (0.597) [0.492, 0.800]	4.24 (0.301) [0.228, 0.406]	4.17 (0.296) [0.237, 0.420]	1.7604 [-35.025, 27.891]
Lingual gyrus	18.01 (1.279) [0.973, 1.420]	8.96 (0.636) [0.465, 0.711]	9.05 (0.643) [0.478, 0.740]	-0.9827 [-23.348, 17.317]
Occipital fusiform gyrus	8.76 (0.622) [0.345, 0.638]	4.32 (0.306) [0.160, 0.329]	4.45 (0.316) [0.159, 0.335]	-2.9758 [-40.799, 35.291]
Inf. occipital gyrus	12.76 (0.906) [0.780, 1.167]	7.49 (0.532) [0.371, 0.603]	5.27 (0.374) [0.372, 0.601]	34.7585 [-27.796, 30.694]
Middle occipital gyrus	12.38 (0.879) [0.632, 1.035]	5.19 (0.369) [0.274, 0.484]	7.19 (0.511) [0.330, 0.579]	-32.3025 [-50.081, 11.149]
Sup. occipital gyrus	7.79 (0.553) [0.310, 0.708]	4.33 (0.308) [0.202, 0.434]	3.45 (0.245) [0.146, 0.320]	22.5483 [-21.140, 48.395]
Occipital pole	4.25 (0.302) [0.172, 0.462]	1.80 (0.128) [0.073, 0.225]	2.45 (0.174) [0.079, 0.257]	-30.8450 [-61.751, 34.472]
**Limbic cortex**	46.20 (3.280) [2.932, 3.810]	20.11 (1.428) [1.450, 1.926]	26.08 (1.852) [1.429, 1.936]	-25.8643 [-15.018, 15.092]
Entorhinal area	3.27 (0.232) [0.224, 0.360]	1.60 (0.113) [0.107, 0.186]	1.68 (0.119) [0.106, 0.184]	-4.8181 [-26.165, 27.671]
Anterior cingulate gyrus	12.51 (0.888) [0.796, 1.223]	5.16 (0.366) [0.372, 0.643]	7.35 (0.522) [0.382, 0.622]	-35.0051 [-33.776, 36.861]
Middle cingulate gyrus	13.50 (0.959) [0.721, 1.019]	5.55 (0.394) [0.349, 0.524]	7.96 (0.565) [0.341, 0.526]	-35.7245 [-25.578, 27.946]
Posterior cingulate gyrus	11.72 (0.832) [0.633, 0.898]	5.44 (0.386) [0.301, 0.457]	6.28 (0.446) [0.317, 0.457]	-14.2651 [-22.214, 17.887]
Parahippocampal gyrus	5.18 (0.368) [0.343, 0.525]	2.36 (0.168) [0.164, 0.296]	2.82 (0.200) [0.170, 0.271]	-17.6288 [-22.252, 16.044]
**Insular cortex**	32.59 (2.314) [2.149, 2.765]	15.41 (1.094) [1.092, 1.423]	17.18 (1.220) [1.040, 1.359]	-10.8330 [-5.297, 14.514]
Anterior insula	9.11 (0.647) [0.577, 0.823]	3.83 (0.272) [0.277, 0.407]	5.27 (0.374) [0.294, 0.422]	-31.6310 [-16.919, 7.267]
Posterior insula	5.03 (0.357) [0.287, 0.435]	2.65 (0.188) [0.146, 0.227]	2.39 (0.169) [0.135, 0.213]	10.3884 [-11.376, 25.330]
Central operculum	9.35 (0.664) [0.549, 0.757]	4.97 (0.353) [0.267, 0.389]	4.38 (0.311) [0.266, 0.384]	12.6905 [-19.822, 22.674]
Frontal operculum	4.63 (0.328) [0.274, 0.432]	1.92 (0.137) [0.128, 0.214]	2.70 (0.192) [0.132, 0.233]	-33.6696 [-88.172, 8.863]
Parietal operculum	4.48 (0.318) [0.291, 0.489]	2.04 (0.145) [0.116, 0.227]	2.44 (0.173) [0.158, 0.279]	-17.8944 [-63.144, 16.284]

CSF	Total ($cm^3/\%$)	Right ($cm^3/\%$)	Left ($cm^3/\%$)	Asymmetry ($\%$)
Inf. Lateral Ventricle	0.72 (0.051) [0.016, 0.112]	0.40 (0.028) [0.003, 0.063]	0.32 (0.023) [0.008, 0.054]	22.3214 [-127.8, 97.820]
Lateral Ventricle	7.31 (0.519) [0.070, 2.864]	3.61 (0.257) [0.042, 1.506]	3.70 (0.263) [0.000, 1.418]	-2.3105 [-53.471, 63.026]
3rd Ventricle	1.08 (0.077) [0.004, 0.132]			
4th Ventricle	2.10 (0.149) [0.060, 0.226]			
External CSF	143.45 (10.185) [3.149, 12.199]			

Cerebellar vermis	Total ($cm^3/\%$)
Lobules I-V	3.97 (0.282) [0.239, 0.406]
Lobules VI-VII	2.10 (0.149) [0.128, 0.213]
Lobules VIII-X	2.74 (0.195) [0.170, 0.271]

Cortical thickness

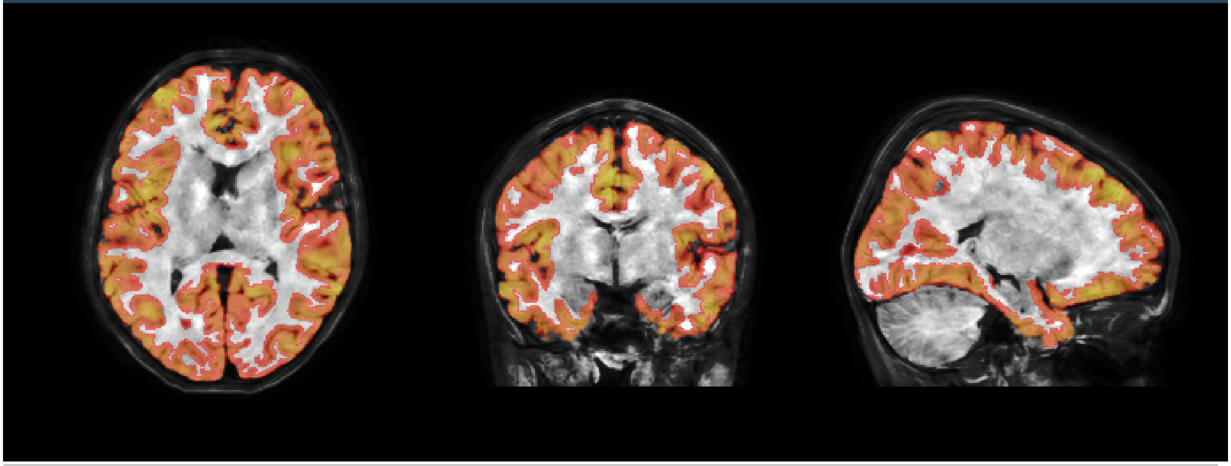

Thickness	Total (mm/norm.)	Right (mm/norm.)	Left (mm/norm.)	Asymmetry (%)
**Frontal lobe**	2.53 (0.023) [0.022, 0.030]	2.32 (0.021) [0.022, 0.030]	2.74 (0.024) [0.022, 0.031]	-16.6618 [-6.881, 6.779]
Frontal pole	2.22 (0.020) [0.022, 0.033]	2.06 (0.018) [0.023, 0.035]	2.36 (0.021) [0.020, 0.032]	-13.3861 [-11.022, 47.317]
Gyrus rectus	2.39 (0.021) [0.021, 0.039]	2.02 (0.018) [0.021, 0.040]	2.75 (0.025) [0.003, 0.030]	-30.5292 [-25.563, 32.598]
Opercular inf. frontal gyrus	2.23 (0.020) [0.020, 0.028]	1.89 (0.017) [0.019, 0.029]	2.62 (0.023) [0.020, 0.029]	-32.2281 [-30.345, 19.659]
Orbital inf. frontal gyrus	2.45 (0.022) [0.022, 0.032]	1.57 (0.014) [0.022, 0.034]	3.11 (0.028) [0.020, 0.031]	-66.0838 [-15.975, 37.133]
Triangular inf. frontal gyrus	2.29 (0.020) [0.021, 0.029]	2.06 (0.018) [0.020, 0.030]	2.51 (0.022) [0.020, 0.030]	-19.8230 [-25.594, 21.825]
Medial frontal cortex	3.13 (0.028) [0.025, 0.037]	2.36 (0.021) [0.025, 0.038]	3.87 (0.034) [0.023, 0.036]	-48.3851 [-15.677, 29.924]
Middle frontal gyrus	2.70 (0.024) [0.023, 0.033]	2.81 (0.025) [0.022, 0.033]	2.57 (0.023) [0.023, 0.033]	8.9782 [-17.130, 13.000]
Anterior orbital gyrus	2.99 (0.027) [0.025, 0.038]	2.38 (0.021) [0.024, 0.039]	3.46 (0.031) [0.024, 0.039]	-36.9746 [-22.064, 23.584]
Lateral orbital gyrus	3.22 (0.029) [0.024, 0.036]	2.65 (0.024) [0.025, 0.039]	3.92 (0.035) [0.022, 0.035]	-38.8179 [-7.373, 36.445]
Medial orbital gyrus	2.79 (0.025) [0.021, 0.035]	2.27 (0.020) [0.019, 0.035]	3.24 (0.029) [0.022, 0.036]	-35.0041 [-27.994, 11.229]
Posterior orbital gyrus	2.85 (0.025) [0.025, 0.039]	1.84 (0.016) [0.025, 0.040]	3.41 (0.030) [0.024, 0.039]	-59.8245 [-15.740, 20.823]
Precentral gyrus	2.33 (0.021) [0.016, 0.023]	2.37 (0.021) [0.016, 0.023]	2.28 (0.020) [0.016, 0.023]	3.8839 [-18.899, 12.234]
Precentral gyrus medial segment	2.44 (0.022) [0.015, 0.025]	2.00 (0.018) [0.016, 0.026]	2.89 (0.026) [0.014, 0.024]	-36.4827 [-11.946, 35.799]
Subcallosal area	2.07 (0.018) [0.013, 0.033]	1.45 (0.013) [0.011, 0.033]	2.56 (0.023) [0.013, 0.034]	-55.2468 [-36.807, 31.399]

Sup. frontal gyrus	2.29 (0.020) [0.021, 0.030]	2.11 (0.019) [0.021, 0.030]	2.45 (0.022) [0.021, 0.030]	-15.0381 [-11.892, 10.268]
Sup. frontal gyrus medial segment	2.73 (0.024) [0.027, 0.036]	2.44 (0.022) [0.027, 0.037]	3.03 (0.027) [0.026, 0.036]	-21.5485 [-16.334, 17.443]
Supplementary motor cortex	2.62 (0.023) [0.022, 0.032]	1.76 (0.016) [0.023, 0.033]	3.39 (0.030) [0.021, 0.032]	-63.3788 [-10.604, 24.325]
**Temporal lobe**	2.82 (0.025) [0.017, 0.025]	2.92 (0.026) [0.018, 0.026]	2.72 (0.024) [0.017, 0.025]	7.2533 [-4.385, 14.506]
Fusiform gyrus	2.98 (0.027) [0.028, 0.040]	2.95 (0.026) [0.027, 0.040]	3.00 (0.027) [0.028, 0.040]	-1.6298 [-13.129, 9.488]
Planum polare	1.55 (0.014) [0.016, 0.027]	1.74 (0.016) [0.017, 0.028]	1.38 (0.012) [0.015, 0.027]	23.3279 [-20.828, 36.342]
Planum temporale	2.04 (0.018) [0.017, 0.027]	2.06 (0.018) [0.016, 0.027]	2.01 (0.018) [0.016, 0.028]	2.4646 [-67.224, 11.247]
Inf. temporal gyrus	2.89 (0.026) [0.027, 0.039]	3.22 (0.029) [0.024, 0.037]	2.56 (0.023) [0.029, 0.041]	22.7696 [-25.460, -2.435]
Middle temporal gyrus	3.08 (0.027) [0.025, 0.035]	3.10 (0.028) [0.025, 0.035]	3.06 (0.027) [0.024, 0.034]	1.3350 [-9.022, 14.436]
Sup. temporal gyrus	2.41 (0.022) [0.021, 0.029]	2.57 (0.023) [0.021, 0.030]	2.24 (0.020) [0.019, 0.029]	13.8140 [-9.860, 25.054]
Transverse temporal gyrus	2.30 (0.021) [0.016, 0.028]	1.79 (0.016) [0.016, 0.028]	2.78 (0.025) [0.016, 0.029]	-43.2894 [-30.157, 24.384]
Temporal pole	3.08 (0.027) [0.030, 0.042]	3.21 (0.029) [0.029, 0.043]	2.95 (0.026) [0.029, 0.042]	8.6557 [-12.449, 15.666]
**Parietal lobe**	2.14 (0.019) [0.026, 0.035]	2.13 (0.019) [0.026, 0.035]	2.15 (0.019) [0.026, 0.035]	-1.0882 [-5.495, 8.572]
Angular gyrus	2.45 (0.022) [0.019, 0.029]	2.49 (0.022) [0.019, 0.029]	2.39 (0.021) [0.020, 0.030]	4.1615 [-27.405, 12.851]
Postcentral gyrus	1.67 (0.015) [0.011, 0.017]	1.87 (0.017) [0.011, 0.017]	1.48 (0.013) [0.011, 0.018]	23.0214 [-23.936, 19.153]
Postcentral gyrus medial segment	1.28 (0.011) [0.007, 0.016]	1.09 (0.010) [0.006, 0.018]	1.46 (0.013) [0.006, 0.016]	-28.6530 [-37.653, 67.074]
Precuneus	2.64 (0.024) [0.020, 0.032]	2.45 (0.022) [0.020, 0.032]	2.81 (0.025) [0.021, 0.032]	-13.9290 [-16.390, 12.017]
Sup. parietal lobule	1.83 (0.016) [0.012, 0.021]	1.44 (0.013) [0.012, 0.020]	2.19 (0.020) [0.013, 0.022]	-41.0887 [-31.150, 9.507]
Supramarginal gyrus	2.10 (0.019) [0.020, 0.029]	2.42 (0.022) [0.019, 0.028]	1.79 (0.016) [0.020, 0.030]	29.9709 [-24.432, 14.417]

**Occipital lobe**	2.15 (0.019) [0.015, 0.026]	2.20 (0.020) [0.015, 0.026]	2.10 (0.019) [0.015, 0.026]	4.5253 [-8.712, 10.416]
Calcarine cortex	1.42 (0.013) [0.009, 0.023]	1.21 (0.011) [0.008, 0.023]	1.56 (0.014) [0.009, 0.024]	-25.1577 [-35.568, 19.796]
Cuneus	1.78 (0.016) [0.011, 0.023]	1.59 (0.014) [0.011, 0.023]	1.98 (0.018) [0.011, 0.024]	-21.3942 [-30.708, 18.708]
Lingual gyrus	2.10 (0.019) [0.017, 0.030]	2.25 (0.020) [0.017, 0.031]	1.95 (0.017) [0.017, 0.030]	14.5699 [-13.129, 15.388]
Occipital fusiform gyrus	2.16 (0.019) [0.016, 0.029]	2.23 (0.020) [0.016, 0.029]	2.10 (0.019) [0.015, 0.029]	5.9772 [-20.005, 24.289]
Inf. occipital gyrus	2.56 (0.023) [0.018, 0.029]	2.67 (0.024) [0.018, 0.028]	2.41 (0.021) [0.018, 0.029]	10.4081 [-19.574, 15.016]
Middle occipital gyrus	2.74 (0.024) [0.018, 0.030]	2.82 (0.025) [0.018, 0.030]	2.69 (0.024) [0.017, 0.031]	4.8538 [-21.069, 20.704]
Sup. occipital gyrus	2.02 (0.018) [0.011, 0.021]	2.10 (0.019) [0.011, 0.022]	1.92 (0.017) [0.010, 0.021]	9.2787 [-19.441, 37.384]
Occipital pole	1.39 (0.012) [0.007, 0.019]	1.12 (0.010) [0.006, 0.020]	1.60 (0.014) [0.007, 0.020]	-35.1499 [-37.138, 34.044]
**Limbic cortex**	3.16 (0.028) [0.027, 0.037]	2.78 (0.025) [0.027, 0.037]	3.46 (0.031) [0.027, 0.037]	-21.9030 [-9.716, 4.981]
Entorhinal area	2.04 (0.018) [0.025, 0.034]	2.31 (0.021) [0.025, 0.035]	1.77 (0.016) [0.024, 0.034]	26.6446 [-14.632, 22.461]
Anterior cingulate gyrus	3.39 (0.030) [0.031, 0.043]	2.88 (0.026) [0.032, 0.044]	3.74 (0.033) [0.030, 0.043]	-25.8862 [-9.194, 15.935]
Middle cingulate gyrus	3.27 (0.029) [0.025, 0.037]	2.70 (0.024) [0.025, 0.037]	3.67 (0.033) [0.024, 0.037]	-30.5515 [-13.514, 16.872]
Posterior cingulate gyrus	3.62 (0.032) [0.026, 0.038]	3.31 (0.030) [0.025, 0.038]	3.88 (0.035) [0.026, 0.038]	-15.8913 [-12.414, 11.182]
Parahippocampal gyrus	2.03 (0.018) [0.019, 0.030]	1.82 (0.016) [0.020, 0.031]	2.21 (0.020) [0.019, 0.030]	-19.2175 [-11.812, 21.250]
**Insular cortex**	2.81 (0.025) [0.024, 0.034]	2.50 (0.022) [0.024, 0.034]	3.09 (0.028) [0.024, 0.034]	-21.2829 [-8.514, 9.811]
Anterior insula	3.29 (0.029) [0.027, 0.038]	2.73 (0.024) [0.027, 0.038]	3.70 (0.033) [0.027, 0.038]	-30.3286 [-11.693, 9.608]
Posterior insula	2.66 (0.024) [0.020, 0.033]	2.47 (0.022) [0.021, 0.034]	2.87 (0.026) [0.020, 0.033]	-14.9536 [-15.156, 21.342]
Central operculum	2.62 (0.023) [0.023, 0.034]	2.37 (0.021) [0.022, 0.034]	2.89 (0.026) [0.023, 0.034]	-19.7160 [-18.905, 14.445]
Frontal operculum	3.08 (0.028) [0.025, 0.036]	2.66 (0.024) [0.024, 0.037]	3.38 (0.030) [0.024, 0.036]	-23.7620 [-52.556, -4.057]
Parietal operculum	2.13 (0.019) [0.018, 0.028]	2.25 (0.020) [0.017, 0.029]	2.03 (0.018) [0.017, 0.029]	9.9464 [-25.967, 25.678]
